# Development of Glatopa® (Glatiramer Acetate): The First FDA-Approved Generic Disease-Modifying Therapy for Relapsing Forms of Multiple Sclerosis

**DOI:** 10.1177/0897190017725984

**Published:** 2017-08-29

**Authors:** Christine Bell, James Anderson, Tanmoy Ganguly, James Prescott, Ishan Capila, Jonathan C. Lansing, Richard Sachleben, Mani Iyer, Ian Fier, James Roach, Kristina Storey, Paul Miller, Steven Hall, Daniel Kantor, Benjamin M. Greenberg, Kavita Nair, Joseph Glajch

**Affiliations:** 1 Analytical Development, Momenta Pharmaceuticals, Inc, Cambridge, MA, USA; 2 Pharmaceutical Sciences, Momenta Pharmaceuticals, Inc, Cambridge, MA, USA; 3 Research and Development, Momenta Pharmaceuticals, Inc, Cambridge, MA, USA; 4 Analytical Chemistry, Momenta Pharmaceuticals, Inc, Cambridge, MA, USA; 5 Research, Momenta Pharmaceuticals, Inc, Cambridge, MA, USA; 6 Complex Generics Manufacturing, Technical Operations, Momenta Pharmaceuticals, Inc, Cambridge, MA, USA; 7 Chemical Development and Manufacturing, Momenta Pharmaceuticals, Inc, Cambridge, MA, USA; 8 Program and Project Management, Momenta Pharmaceuticals, Inc, Cambridge, MA, USA; 9 Clinical Development and Regulatory Affairs, Momenta Pharmaceuticals, Inc, Cambridge, MA, USA; 10 Regulatory Affairs, Momenta Pharmaceuticals, Inc, Cambridge, MA, USA; 11 Medical Affairs and Communications, Momenta Pharmaceuticals, Inc, Cambridge, MA, USA; 12 Medical Affairs, Sandoz, Inc, a Novartis Division, Princeton, NJ, USA; 13 Division of Neurology, Florida Atlantic University, Boca Raton, FL, USA; 14 Neurology and Neurotherapeutics, Pediatrics, UT Southwestern Medical Center, Dallas, TX, USA; 15 Department of Clinical Pharmacy, University of Colorado School of Pharmacy, Aurora, CO, USA

**Keywords:** disease-modifying therapy, generic drugs, glatiramer acetate, multiple sclerosis

## Abstract

The multiple sclerosis (MS) treatment landscape in the United States has changed dramatically over the past decade. While many disease-modifying therapies (DMTs) have been approved by the US Food and Drug Administration (FDA) for the treatment of relapsing forms of MS, DMT costs continue to rise. The availability of generics and biosimilars in the MS-treatment landscape is unlikely to have a major impact on clinical benefit. However, their availability will provide alternative treatment options and potentially lower costs through competition, thus increasing the affordability of and access to these drugs. In April 2015, the first generic version of the complex drug glatiramer acetate (Glatopa® 20 mg/mL) injection was approved in the United States as a fully substitutable generic for all approved indications of the 20 mg/mL branded glatiramer acetate (Copaxone®) dosage form. Despite glatiramer acetate’s complex nature—being a chemically synthesized (ie, nonbiologic) mixture of peptides—the approval occurred without conducting any clinical trials. Rather, extensive structural and functional characterization was performed to demonstrate therapeutic equivalence to the innovator drug. The approval of Glatopa signifies an important milestone in the US MS-treatment landscape, with the hope that the introduction of generic DMTs and eventually biosimilar DMTs will lead to future improvements in the affordability and access of these much-needed treatments for MS.

## Introduction

Multiple sclerosis (MS) is a chronic inflammatory demyelinating disease of the central nervous system affecting more than 2 million individuals globally and approximately 400 000 in the United States.^1,2^ Relapsing forms of MS include clinically isolated syndrome (CIS; a first demyelinating episode), relapsing-remitting MS (RRMS), secondary-progressive MS, and progressive-relapsing MS.^1-3^ The symptoms of MS can be highly variable, both in severity and duration, but may include visual disturbances, bladder/bowel dysfunction, weakness, impaired balance, vertigo, numbness, tingling, pain, and cognitive dysfunction.^1,2^

The past decade has seen a dramatic change in the US MS-treatment landscape. While no cure for MS is currently available, treatment options exist to reduce the frequency of relapses, manage symptoms, and slow disease progression. Many US Food and Drug Administration (FDA)–approved disease-modifying therapies (DMTs) are currently available in the US market for patients with relapsing forms of MS, including interferon beta-1b (Betaseron®^4^, Extavia®^5^), interferon beta-1a (Avonex®^6^ and Rebif®^7^), glatiramer acetate (Copaxone^8^), natalizumab (Tysabri®^9^), fingolimod (Gilenya®^10^), teriflunomide (Aubagio®^11^), dimethyl fumarate (Tecfidera®^12^), alemtuzumab (Lemtrada®^13^), peginterferon beta-1a (Plegridy®^14^), and daclizumab (Zinbryta™^15^).^16-20^ In general, the currently available medications primarily target the mechanisms that underlie inflammation. Early and ongoing treatment helps to minimize early inflammation, reduce damage in nerve fibers (axons), and reduce loss of brain tissue.^2^ The anti-inflammatory effects of these agents are largely believed to result from the inhibition of T-lymphocyte proliferation, from a shift of the cytokine response from an inflammatory response to an anti-inflammatory profile, and/or from a reduction in the migration of inflammatory cells across the blood–brain barrier.^17^ More recently, ocrelizumab (Ocrevus™) became the first and only DMT approved for the treatment of primary progressive and relapsing forms of MS.^21^ Although the precise mechanism by which ocrelizumab exerts its therapeutic effects in MS is unknown, it is presumed to involve the depletion of pre-B and mature B lymphocytes.^21^

Glatiramer acetate continues to be a valuable first-line treatment option in relapsing forms of MS given its extensive clinical trial and “real-world” efficacy and safety data.^22,23^ Furthermore, Glatopa, a generic version of glatiramer acetate, was subsequently developed; Glatopa received FDA approval in 2015, making it the first approved substitutable DMT for MS (in particular, for relapsing forms of MS).^24^

This article will provide an overview of the scientific processes involved in the development of generic glatiramer acetate that resulted in FDA approval and highlight the FDA’s framework for the demonstration of equivalence.

## Copaxone—A Brief History

Glatiramer acetate, also known as copolymer-1, is a heterogeneous mixture of peptides comprising 4 amino acids and is similar to myelin basic protein.^22,23^ Copolymer-1 was first discovered in the late 1960s during research to produce an antigen capable of inducing experimental autoimmune encephalomyelitis (EAE), an animal model of MS.^25^ However, instead of inducing EAE, copolymer-1 immunization of EAE animal models was shown to protect against EAE induction and to reduce symptoms of established EAE.^25-27^ The exact mechanism of glatiramer acetate is unknown, but it is thought to act by modifying immune processes that are believed to be responsible for the pathogenesis of MS.

To date, glatiramer acetate is the only therapeutic peptide approved for the treatment of MS. Subcutaneous glatiramer acetate 20 mg/mL was approved by the FDA in 1996, and the 40 mg/mL triweekly dose was approved in 2014 for the treatment of patients with relapsing forms of MS.^8^ The approval of these 2 versions of glatiramer acetate was supported by data from 5 placebo-controlled trials (4 trials using a 20 mg/mL/d dose; 1 trial using a 40 mg/mL triweekly dose).^28-32^ In controlled studies of Copaxone, the most common adverse reactions were injection site reactions, vasodilation, rash, dyspnea, and chest pain.^8^ A head-to-head comparison trial between dimethyl fumarate and glatiramer acetate reported numerically (but not statistically significantly) better effect from dimethyl fumarate.^33^ Head-to-head comparisons of glatiramer acetate and subcutaneous high-dose and high-frequency interferon beta-1a and interferon beta-1b have shown similar clinical benefit, with some magnetic resonance imaging parameters favoring the interferon beta preparations.^34,35^

## Approval of Generic Drugs in the United States—The Abbreviated New Drug Application Pathway

In mid-2016, approximately 80% of US prescriptions were for generic drugs.^36^ The FDA defines a generic drug product as “one that is comparable to an innovator drug product in dosage form, strength, route of administration, quality, performance characteristics, and intended use.”^24^ The approval of generics through the Abbreviated New Drug Application (ANDA) pathway was established by the Drug Price Competition and Patent Term Restoration Act of 1984 (also known as the Hatch-Waxman Act), which permits FDA approval of applications to market generic versions of brand-name drugs without the need for costly and duplicative clinical trials, thus expediting the availability of less costly generic drugs.^37^ Unlike the standard FDA drug-approval process that requires extensive preclinical (animal) and clinical (human) testing to establish safety and effectiveness,^38^ the approvals of generic drug products via the ANDA pathway do not generally require inclusion of preclinical and clinical data.^39^ Instead, physicochemical equivalence and bioequivalence to the innovator drug must be demonstrated scientifically to establish therapeutic equivalence,^39-41^ although the FDA generally waives the need to demonstrate bioequivalence for injectable drug products. According to 21CFR320.22, the FDA’s guidance document on bioavailability and bioequivalence requirements,^42^ the bioequivalence of an injectable generic product (eg, Glatopa) may be considered self-evident if it is a parenteral solution intended solely for administration by injection and contains the same active and inactive ingredients in the same concentrations as the approved drug product (eg, Copaxone). Taken together, the underlying premise for approval of a generic via the ANDA pathway is that the generic drug product can be used to substitute the innovator drug product with the full expectation that they will have the same efficacy and safety profile.

It is important to note that glatiramer acetate is chemically synthesized, which differs from biological drugs that are produced in living cell cultures (ie, natalizumab, alemtuzumab, daclizumab, and ocrelizumab).^9,13,15,21^ As a result, generic drugs and biosimilars (the latter also known as follow-on biologics) are faced with different challenges and are subjected to different FDA-approval requirements. The biotechnology used to produce biologics differs from chemically produced drugs in the nature, amount, and ability to control the profiles of impurities and related substances. For generic drug products approved via the ANDA pathway, active ingredients must be shown to be the same, with the same dosage form, concentration, and bioequivalence as that of the approved drug product.^40^ The generic drug is determined to be therapeutically equivalent to the approved drug product if these requirements are met, thus avoiding the need for clinical trials to be performed. In contrast, for biosimilars, the active ingredients need only be “highly similar” to that of the FDA-approved reference biologic drug product; to date, clinical trials have been part of the process to establish equivalence.^43,44^

In 2015, the FDA approved the first generic version of glatiramer acetate, Glatopa, as a fully substitutable injectable aqueous solution (AP)-rated generic for all approved indications of the Copaxone 20 mg/mL dosage form.^24^ Glatiramer acetate is classified by the FDA as a complex drug mixture of peptides and was therefore eligible for approval via the ANDA pathway. The FDA indicated that glatiramer acetate is manufactured by a chemical process from small molecules and is not considered a protein because it does not have “a defined and specific amino acid sequence.”^45^ Glatiramer acetate is best described as a heterogeneous mixture of amino acid copolymers.^45^ Although citizen petitions made claims that the drug product formulation of Copaxone is not a true solution,^45^ the Copaxone formulation is described as a clear solution in the drug product package insert,^8^ and the FDA asserted that there was sufficient supportive evidence from physicochemical characterization studies provided in the Glatopa filing to indicate that glatiramer acetate is fully dissolved as in a true solution.^45^ The complexity of the glatiramer acetate peptide mixture makes definitive characterization by analytical methodology challenging, but it is possible to characterize product-specific attributes using modern analytical and quantitative analysis methods, thus enabling sameness of the active ingredient(s) to be established between the innovator drug (Copaxone) and the generic drug product (Glatopa).^46^ Glatiramer acetate is developed using a well-controlled, robust manufacturing process that in turn allows the use of physicochemically synthesized, reverse engineering of the synthetic process as 1 component in the demonstration of equivalence between Glatopa and Copaxone.

## Framework to Evaluate Equivalence—A Focus on Glatiramer Acetate

On April 16, 2015, the FDA denied a citizen petition requesting that any ANDA that references Copaxone not be approved, and indicated that it would be approving an ANDA for a generic version of glatiramer acetate (ie, Glatopa) that references Copaxone.^45^ Within this denial letter, the FDA provided a comprehensive explanation detailing the framework with which they evaluated equivalence, along with responses to issues raised in the citizen petitions.^45^ This framework included an in-depth evaluation of the analytical scientific approach undertaken to address the 4 major criteria required to establish equivalence between generic and branded glatiramer acetate injection, along with the FDA’s own analytical testing to confirm these criteria for demonstration of sameness.

The characterization of glatiramer acetate was accomplished through a thorough understanding of the chemistry, manufacturing process, and biological effects of glatiramer acetate, and that involved a detailed characterization of the active ingredient and development of a process that reproducibly yields an equivalent material. While this can be simply achieved through first-principle chemical analysis for small-molecule generics, glatiramer acetate is a mixture of synthetic polypeptides with variable molecular weights and sequences, manufactured from copolymerization of l-alanine, l-glutamic acid, l-lysine, and l-tyrosine, thus requiring analytical approaches that provide comprehensive data on how the manufacturing process used to create glatiramer acetate defines its product-specific attributes.^47^ This was supported by the FDA, which acknowledged that while there is no single physicochemical or biological characterization technique to establish active ingredient sameness between the generic glatiramer acetate product (Glatopa) and the reference listed drug (Copaxone 20 mg/mL), combined evidence from a range of scientific methods may be used to demonstrate equivalence based on 4 major criteria: (1) equivalence of fundamental reaction scheme; (2) equivalence of physicochemical properties, including composition; (3) equivalence of structural signatures for polymerization and depolymerization; and (4) equivalence of activity in functional biological assays (see Table 1).^45^ Therefore, the FDA evaluated data submitted as part of the ANDA application, which was obtained from extensive physicochemical, biological, and immunological characterization techniques (>60 methods) that were undertaken to characterize glatiramer acetate and demonstrate sameness between Glatopa and Copaxone.^45^

**Table 1.** Proposed Model Framework for Establishing Active Ingredient Sameness Between Generic Glatiramer Acetate (Glatopa) and Branded Glatiramer Acetate (Copaxone) Based on 4 Major Criteria.^29,31^

Framework Criteria	Definition of Criteria	Operationalization
Fundamental reaction scheme	The active ingredient of Glatopa must be produced by an equivalent fundamental reaction scheme using the same (or equivalent) starting materials, reagents, and basic chemical steps as Copaxone	Starting materials 4 amino acid NCAs Polymerization initiator (diethylamine) Reagents Chemical reagent(s) for acid-catalyzed cleavage conditions (eg, HBr) Basic chemistry Polymerization of NCAs with an initiator to yield an intermediate copolymer Partial depolymerization and deprotection of the initially formed protected peptide mixture Final deprotection of the second intermediate peptide mixture and purification
Physicochemical properties (including composition)	The physicochemical properties of Glatopa and Copaxone must be equivalent to confirm Active ingredient sameness at a greater level of quantitative detail Equivalence of underlying reaction processes	Amino acid building blocks Determination of amino acid content by complete hydrolysis of the peptide mixture to its amino acid components (l-glutamic acid, l-lysine, l-alanine, l-tyrosine) Measurement of amino acid chirality Molecular weight distribution Comparison of molar mass moments and polydispersity using size exclusion chromatography, mass spectroscopy, or other appropriate methods Spectroscopic fingerprints Overall properties of the drug measured by spectroscopic methods (eg, NMR, FT-IR) Circular dichroism measure to determine the distribution of secondary structure of peptide chains (eg, α-helical content)
Structural signatures for polymerization and depolymerization	Product-specific attributes that are directly attributable and sensitive to the chemical processes of polymerization and depolymerization	Structural signatures for polymerization initiation Amino acid-initiator proportions Total initiator content in copolymer Structural signatures for propagational shift during polymerization N-terminal amino acid sequence Structural signatures for cleavage during partial depolymerization N- and C-terminal amino acid proportions Ratio of “uncapped” versus “capped” C-termini
Functional biological assays	Assessment of GA biological functions to demonstrate the equivalence of Glatopa and Copaxone with regard to aggregate biological function and key aspects of its biology	Biochemical assays (in vitro and in vivo) APC biology (THP-1 chemokine assay) T-cell biology (generation of murine Th2 polarized T cells; murine Th2 polarized T cell IL-4 ELISA) B-cell response (anti-GA antibody response) Assessment of aggregate biology using EAE animal models

Abbreviations: APC, antigen presenting cell; EAE, experimental autoimmune encephalomyelitis; ELISA, enzyme-linked immunosorbent assay; FT-IR, Fourier transform infrared spectroscopy; GA, glatiramer acetate; IL-4, interleukin-4; NCA, N-carboxyanhydride; NMR, nuclear magnetic resonance imaging; THP-1, human monocytic cell line.

**Figure 1.**
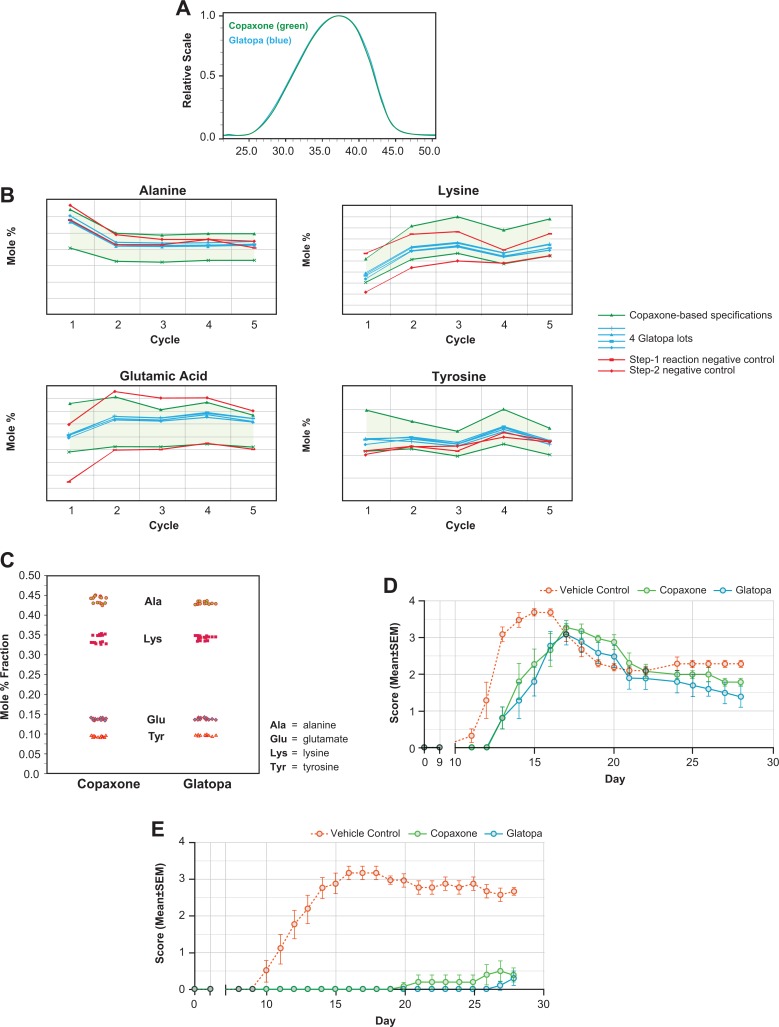
Examples of structural and biological analyses used to establish equivalence of Glatopa and Copaxone 20 mg/mL, which includes assessment of (A), molar mass distributions; (B), amino acid levels for the first 5 cycles of N-terminal analysis by Edman degradation; (C), total amino acid composition; (D), the proteolipid peptide version of the experimental autoimmune encephalomyelitis (EAE) prophylaxis model; and (E), the myelin oligodendrocyte glycoprotein version of the EAE prophylaxis model.

Firstly, equivalence of the fundamental reaction scheme used to produce the active ingredient of generic glatiramer acetate injection was demonstrated using the same starting materials (ie, 4 amino acid N-carboxyanhydrides), reagents (ie, polymerization initiator and chemical reagents for acid-catalyzed cleavage conditions), and basic chemical steps (ie, polymerization, depolymerization and deprotection, and purification) as reported for Copaxone.^47^ Second, a range of methods (approximately 45) were utilized to characterize the physicochemical properties of glatiramer acetate, thus providing a demonstration of active ingredient sameness between Glatopa and Copaxone.^47^ Several examples of physicochemical equivalence are provided in Figure 1, including similar molar mass distributions of Glatopa and Copaxone, as measured by size exclusion chromatography (Figure 1A), and similar amino acid compositions as mole fractions of final glatiramer acetate for several lots of Glatopa and Copaxone (Figure 1B). Third, process characterization studies were conducted to identify structural/process signatures for each step during the chemical synthesis of glatiramer acetate, including polymerization initiation (eg, amino acid diethylamide proportions), propagational shift during polymerization (eg, relative amino acid levels at the N-termini of glatiramer acetate for the first 5 cycles of N-terminal analysis by Edman degradation, with Glatopa lots contained within acceptance criteria based on the observed range for multiple lots of Copaxone [Figure 1C; also shown are 2 negative controls]), and cleavage reactions in partial depolymerization (eg, N- and C-terminal amino acid proportions); equivalence of these structural/process signatures between Glatopa and Copaxone indicates that the processes involved in the manufacture of these two drugs are equivalent.^47^ Finally, the fourth major criterion was used to confirm equivalence and involved the employment of multiple confirmatory and orthogonal biological/immunological assays, including major histocompatibility complex II class binding, antigen-presenting cell function, T-cell proliferation, T-cell polarization, B-cell biology, antibody response, immunorecognition, anti-inflammatory effects, and neuroprotection.^47^ These assays were performed in well-established animal models of MS (eg, EAE) and not in humans, with findings showing the ability of Glatopa and Copaxone to provide equivalent delays in symptom onset, disease intensity, and antibody response (Figure 1D). Specifically, EAE was induced with immunogenic myelin neuroantigens, directly by immunization with proteolipid peptide (PLP_139–151_; a simulation of RRMS; Figure 1D) or neuroantigen myelin oligodendrocyte glycoprotein (MOG_35–55_; a simulation of primary progressive MS, Figure 1E). In both versions, Glatopa and Copaxone delayed symptom onset and reduced the magnitude of disease intensity, with no statistically significant differences (*P* < .05) between Glatopa and Copaxone. In addition to EAE models, gene expression profiling conducted in glatiramer acetate–responsive mouse Th2 polarized T cells did not show any significant differences in gene expression profiles (eg, chemokine and interleukin expression levels) between Glatopa and Copaxone.^48^

In addition to the evaluation of available data characterizing generic glatiramer acetate and Copaxone to establish equivalence based on the 4 major criteria discussed above, the FDA conducted its own analytical testing of generic glatiramer acetate and Copaxone to confirm these sameness criteria.^45^ Quantitative characterization could not distinguish generic glatiramer acetate from Copaxone, while glatiramer acetate–like products marketed outside of the United States and negative controls were clearly distinguished from Copaxone.^45^ More specifically, the quantitative characterization of generic glatiramer acetate is consistent with the same characterization of Copaxone, taking into account batch-to-batch variability.^45^

Taken together, the FDA’s citizen petition denial letter and published data to date showcase a framework that may be employed to “deconstruct” the complexity of glatiramer acetate through an extensive multitude of physicochemical, biological, and immunological techniques. These techniques enable the characterization of this complex drug mixture, thus allowing comparisons to be made between generic and branded versions to establish therapeutic equivalence.

## Conclusions and Future Perspectives

In the United States, MS DMT costs continue to rise at an alarming rate despite the availability of more than a dozen treatment options, with the cost of first-generation DMTs increasing from $8000 to $11 000 annually per patient at the time of their introduction to approximately $80 000 annually per patient.^49,50^ Approval of the first generic version of glatiramer acetate, Glatopa, signifies an important milestone in the US MS-treatment landscape. While the availability of a single generic in the MS-treatment landscape is unlikely to have a major impact on clinical benefits, the approval of Glatopa paves the path for the development and incorporation of additional generic DMT drug products and biosimilars into the armamentarium for MS. This will provide alternative treatment options and potentially lower costs through competition, thus increasing the affordability and access of these much-needed treatments for MS. Comparisons of historical cost data for branded DMTs with those of Glatopa and other future generics and biosimilars will be of great interest to determine the cost impact of these generic drug products on the DMT market.

Glatopa is manufactured in the United States and has been available since June 2015. The estimated annual cost savings for Glatopa versus the Copaxone 20 mg/mL dose from January 1, 2016, to present is apparent (approximately $17 000 annually per patient based on US wholesale acquisition costs),^50^ but the impact of a future generic version of Copaxone 40 mg/mL on the glatiramer acetate market should also be taken into consideration.^51^ Teva reported that its first-quarter 2016 sales of the Copaxone 40 mg/mL dose accounted for more than 81% of overall Copaxone prescriptions in the United States,^52^ suggesting the conversion of most patients receiving the Copaxone 20 mg/mL dose. Multiple ANDAs for a 40 mg/mL triweekly dose of a Copaxone generic are currently under active review by the FDA, including a 40 mg/mL formulation of Glatopa.

Patient support programs for Glatopa (GlatopaCare®) and Copaxone (Copaxone’s Shared Solutions®) have been established for patients with relapsing forms of MS, their families and caregivers, and physicians.^53,54^ These support programs offer financial support, personalized injection training, ongoing nurse support, and educational resources. For example, injection training is available online and 1-on-1 in-home with a trained nurse to teach patients how to administer Glatopa or Copaxone. Injection devices for Glatopa (Glatopa*ject*) and Copaxone (auto*ject*®2) are both reusable autoinjectors to be used with a prefilled syringe containing the drug.

In summary, FDA approval of Glatopa proves that a rigorous scientific approach and thorough characterization can successfully establish equivalence for complex drugs. The experience gained with Glatopa also provides a framework for the development and approval of future generic MS drug products in the United States. Although currently available DMTs reduce the frequency of relapses, manage symptoms, and slow disease progression, the introduction of generic DMTs will lead to future improvements in the affordability and access of these much-needed treatments for MS.
